# Antimicrobial Stewardship in Ireland (2010-2021): a scoping review protocol

**DOI:** 10.12688/hrbopenres.13917.1

**Published:** 2024-07-31

**Authors:** Fiona Barry, Maura P Smiddy, Anthony P Fitzgerald, Eilis J. O’Reilly, Olive Murphy

**Affiliations:** 1 School of Public Health, University College Cork, Cork, Cork, T12XF62, Ireland; 2 School of Mathematical Sciences and School of Public Health, University College Cork, Cork City, Cork, Cork, T12XF62, Ireland; 3 Bons Secours Health System, College Road, Cork, Cork, T12 DV56, Ireland

**Keywords:** antimicrobial stewardship, Ireland, scoping review

## Abstract

**Background:**

Antimicrobial stewardship programmes (ASP) are essential in promoting responsible antimicrobial use, reducing antimicrobial resistance (AMR) and health care-associated infections. In 2009 the Strategy for the Control of Antimicrobial Resistance in Ireland (SARI), Hospital Antimicrobial Stewardship Working Group published guidance on antimicrobial stewardship (AMS) in hospitals. This paper presents a protocol for a scoping review which aims to examine the current literature to evaluate progress related to the implementation of the SARI (2009) guidance on antimicrobial stewardship in hospitals in Ireland.

**Methods:**

This scoping review will be conducted in line with the five-stage methodological framework by Arksey & O’Mally 2005. We will search the following databases (PubMed, CINAHL, Embase, Web of Science) and targeted websites for articles and reports for possible inclusion in our review. Studies pertaining to AMS undertaken or related to the Republic of Ireland from January 2010 until December 2021 will be included. We will include all study designs. We will map all selected publications to the recommendations of the SARI (2009) guidance document. The protocol follows the guidance of Peters *et al*., 2022. Two reviewers will independently screen studies and reports to assess eligibility with any discrepancies resolved by consensus discussion with a third reviewer.

**Results:**

These will be reported in line with the Preferred Reporting Items for Systematic Reviews and Meta-analyses extension for Scoping Reviews (PRISMA-ScR)and this checklist will be included when the scoping review is published.

**Conclusion:**

This scoping review will map studies and reports to evaluate AMS in relation to national guidelines without restriction on study design or outcomes in the Republic of Ireland. This information has the potential to provide a valuable resource for the implementation of future AMS research and interventions.

## Introduction

The escalating threat posed to human life by antimicrobial resistance (AMR) has intensified over the last 20 years primarily due to the use, abuse, and excessive consumption of antimicrobials agents- antifungals, antiparasitic, antivirals and antibiotics ^1– 2^. These drugs prevent and treat infections, however the global emergence of resistance has compromised their efficacy ^3^.

 Antimicrobial stewardship (AMS) is a methodical approach targeted at ensuring the appropriate use of antimicrobials thereby mitigating the emergence of resistance ^4^.

Antimicrobial Stewardship Programmes (ASP) have been shown to be crucial component of this effort ^5^.

Ireland’s first national document on AMR was published in 2001 ^6^. Specific guidelines on AMS in hospitals were most recently published in 2009 ^7^ by the Strategy for the Control of Antmicobial Resistance (SARI) Committee, reporting to the Health Service Executive and comprised a multidisciplinary team of clinical and academic experts. The guidelines ^7^ summarised recommendations across four key categories: Structure and Organisation, Role of Prescribers, AMS Interventions, and Non acute Residential Healthcare Institutions ^7^.

The aim of this review is to document the progress in AMS implementation in Ireland, mapping the findings to the SARI 2009 ^7^ recomendations.

## Study rationale

To date, the implementation of AMS has not been evaluated in the Irish healthcare setting.

## Dissemination

The results will be shared with AMS teams, presented at conferences, and published as a scoping review.

## Methods

Scoping reviews are conducted when there is extensive evidence on a topic involving academic publications, grey literature, and includes other sources ^8^. This protocol is reported in accordance with JBI Best practice guidelines and reporting items for the mdevelopment of scoping reviews protocols ^9^.

The review process will involve five steps: “(1) identifying the research question; (2) identifying relevant studies; (3) selecting studies; (4) charting the data; (5) collating, summarising and reporting the results” ^10 (p1)^

### Stage 1: Identifying the research question

The research question was identified by asking what progress has been made in relation to the implementation of AMS interventions in healthcare settings in Ireland since the publication of the SARI report in 2009.

***The research objectives***

1. To present evidence of the progress of AMS implementation since the publication of the guidelines ^7^.

2. To identify systems that facilitate or hinder the successful implementation of AMS guidelines from publication in 2009 to 2021.

### Stage 2: Identifying the relevant studies

Based on our research question, we will explore academic databases and grey literature targeting organisations’ websites. Following discussions among all reviewers and a research librarian, we have identified four academic databases: Embase (Elsevier), PubMed, CINHAL, and Web of Science (Clarivate) to search.

 Initially, one reviewer performed a preliminary search to identify common terms and headings, which were then refined according to the focus of this review. We will employ Boolean operators to combine keywords for a more targeted search and examine the reference lists of our chosen studies for potential inclusion. Authors will be contacted if further information about their studies is required.

**Inclusion Criteria:** studies on antimicrobial stewardship focussing on antibiotics; studies originating or relevant to Ireland; literature published in the English language; publications from 01 January 2010 to 31 December 2021. There are no limitations on study designs.

**Exclusion Criteria:** studies on antimicrobial stewardship focussing on other antimicrobials; studies outside Ireland; conference abstracts, editorials theses **,** reviews, and opinion pieces.

***Grey literature search***

Based on our research question asked and keywords previously identified we will search the following targeted websites for publications from January 2010–December 2021.

- The Health Information and Quality Authority (HIQA),
- The Health Service Executive (HSE)
- The Health Protection Surveillance Centre (HPSC)
- The European Centre for Disease Prevention and Control (ECDC)
- The World Health Organization (WHO) publications related to AMS

Once our search is complete all selected studies and reports that meet our inclusion criteria will be downloaded to Mendeley ^11^ and duplicates removed.

### Stage 3: Study selection

The selected title and abstracts will be uploaded to the online tool Covidence ^12^ for screening and retrieve full texts of publications. The reasons for exclusion based on our criteria will be documented in our review. Two reviewers (FB and EOR) will independently undertake screening and any queries that arise will be facilitated by further discussions involving an additional reviewer (OM). Full texts of remaining publications will then be screened for inclusion.

### Stage 4: Charting the data

The following information will be extracted and entered into an MS Excel database ^13^: authors, study details year, objectives, research methods, study populations and findings that are aligned to the SARI 2009 recommendations. Any deviations to the original proposal will be documented in the methods section of the review.

### Stage 5: Collating, summarising, reporting the results

The selected studies will be mapped to the 2009 SARI recommendations ^7^ and the PRISMA-ScR guidelines will be used to report the findings ^14^. The screening process will be presented in a PRISMA flow chart. Due to the broad nature of the research question, a quality appraisal is not essential for this scoping review ^9^.

## Discussion

To our knowledge, this is the first study to map peer reviewed studies and reports to the four recommendations of the first national AMS strategy in order to document progress.

## Ethics and consent

Ethical approval and consent were not required

## Data Availability

No data are associated with this article
