# Supplementary material for: Antimicrobial Stewardship in Ireland (2010-2021): a scoping review protocol
Source: HRB Open Res. 2024 Jul 31;7:52. [Version 1] doi: 10.12688/hrbopenres.13917.1 (PMC11836560; doi:10.12688/hrbopenres.13917.1)
Supplement: Peer review history

# Reviewer report: Reviewer response for version 1

**DOI:** 10.21956/hrbopenres.15266.r42350

**Published:** 2024-09-28

**Authors:** Direk Limmathurotsakul

**Affiliations:** 1 Microbiology, Mahidol-Oxford Tropical Medicine Research Unit, Faculty of Tropical Medicine, Mahidol University, Bangkok / กรุงเทพมหานคร, Thailand

The article by Barry et al is a scoping review protocol evaluating AMS implementation in Ireland. The review will focus on studies relevant to Ireland. The availability of the datasets is not appliable as this is a study protocol. The article and protocol are well-written. 

 I have few minor comments for consideration. 

 1) The primary objective of the scoping review is to evaluate evidence of the 'progress' of AMS implementation. It would be clearer if the authors described potential indicators or outcomes, models or theories they will use to monitor the 'progress'. For example, will authors primarily use a set of structural indicators, process indicators and outcome indicators for AMS? or will they include other indicators to monitor the progress? Although the study aims to be broad due to the nature of the research question, providing a list of potential methods to be used could make the methods clearer and allow for replication by others.

 2) The second objective of the scoping review is to identify ‘systems’ that facilitate or hinder the successful implementation of AMS guidelines. It would be clearer if the authors stated which models or theories the review will use to identify and categorize barriers and enablers to implementation. The authors use the word ‘systems’, but it is unclear whether they will include all domains of potential barriers and facilitators to AMS implementation or focus on only a few domains (e.g., focusing only on environmental context and resources, and behavioral regulation, but not collecting data on studies evaluating social influence on AMS).

 3) In ‘Stage 5’, the authors state that they aim not to use a quality appraisal due to the broad nature of the research question. While this approach may be acceptable, it would still be beneficial to conduct a quality appraisal for any studies/literature that are possible and provide that information to the readers. For studies that cannot be appraised, they can be noted as ‘not applicable’ and provide reasons why it is not possible. This approach would allow readers to understand the quality of the studies included in the review. If none of the studies/literature being reviewed can be appraised for quality, it would still be helpful to provide individual reasons for each study/literature to the readers.

Is the study design appropriate for the research question?

Yes

Is the rationale for, and objectives of, the study clearly described?

Yes

Are sufficient details of the methods provided to allow replication by others?

Yes

Are the datasets clearly presented in a useable and accessible format?

Not applicable

Reviewer Expertise:

Infectious Diseases, Tropical Infectious Diseases, Antimicrobial resistance

I confirm that I have read this submission and believe that I have an appropriate level of expertise to confirm that it is of an acceptable scientific standard, however I have significant reservations, as outlined above.

# Response

**Published:** 2024-12-12

**Authors:** Fiona Barry

**Affiliations:** School of Public Health, University College Cork, Cork, County Cork, Ireland

**Thank you for reviewing this protocol. Please see the responses to your comments below:**

**The primary objective of the scoping review is to evaluate evidence of the 'progress' of AMS implementation. It would be clearer if the authors described potential indicators or outcomes, models or theories they will use to monitor the 'progress'. For example, will authors primarily use a set of structural indicators, process indicators and outcome indicators for AMS? or will they include other indicators to monitor the progress? Although the study aims to be broad due to the nature of the research question, providing a list of potential methods to be used could make the methods clearer and allow for replication by others.**

**Response:** Thank you. To clarify how progress will be ascertained, we will add the following text:

 This paper presents a protocol for a scoping review which describes the progress of the implementation of the recommendations of the 2009 National Strategy for Antimicrobial Resistance in Ireland report.  Papers or reports on AMS in Ireland will be mapped to the list of recommendations.  These recommendations are detailed in the introduction.

**The second objective of the scoping review is to identify ‘systems’ that facilitate or hinder the successful implementation of AMS guidelines. It would be clearer if the authors stated which models or theories the review will use to identify and categorize barriers and enablers to implementation. The authors use the word ‘systems’, but it is unclear whether they will include all domains of potential barriers and facilitators to AMS implementation or focus on only a few domains (e.g., focusing only on environmental context and resources, and behavioural regulation, but not collecting data on studies evaluating social influence on AMS).**

**Response:** **Thank you. Objective 2:** We have reworded objective 2 to reflect that we are not engaging in a full review to find the barriers and facilitators of implementation. The new wording is as follows** *“To explore factors in the included studies that may have supported or hindered progress.”*

 The overall evidence will be mapped to four recommendation categories in the 2009 National Strategy for Antimicrobial Resistance in Ireland with a targeted summary of achievements and challenges per category.  

**In ‘Stage 5’, the authors state that they aim not to use a quality appraisal due to the broad nature of the research question. While this approach may be acceptable, it would still be beneficial to conduct a quality appraisal for any studies/literature that are possible and provide that information to the readers. For studies that cannot be appraised, they can be noted as ‘not applicable’ and provide reasons why it is not possible. This approach would allow readers to understand the quality of the studies included in the review. If none of the studies/literature being reviewed can be appraised for quality, it would still be helpful to provide individual reasons for each study/literature to the readers.**

**Response:  **Thank you. 

 While we agree, this is a scoping review and acceptable not to undertake a quality appraisal. However, we anticipate many of our sources will be from the grey literature and therefore not suitable for appraisal.

# Reviewer report: Reviewer response for version 1

**DOI:** 10.21956/hrbopenres.15266.r41979

**Published:** 2024-08-28

**Authors:** Michael J Durkin

**Affiliations:** 1 Washington University School of Medicine, St. Louis, Missouri, USA

This manuscript is a scoping review protocol to evaluate antimicrobial stewardship programs in Ireland between 2010 and 2021. The authors describe a detailed study protocol with sound methodology. I only have a few comments.

 1) introduction 3rd paragraph last sentence. Reference 7 is listed at the beginning and end of the sentence. 

 2) This scoping review seems to focus on peer reviewed publications to evaluate the progress of antimicrobial stewardship programs in Ireland. Is this the best way to track progress in AMS implementation? In the US we will send out surveys to see if hospitals are meeting CDC recommendations. This may be an alternative approach to supplement published findings as well.

 3) I like the research objectives. It would be nice to have a table or something that outlines what the guidelines have included in 2009. I imagine you all will want to see where places are falling short of the guidelines.

 4) I like objective 2 as well. Some of the implementation science people will use the term facilitators and barriers to successful implementation of... You're probably looking at qualitative studies mostly here. Although I guess discussion sections of manuscripts will work well too. Again, this may also work well by being supplemented with additional survey or interview data. There might be reporting biases in publications with unsuccessful projects that may not have worked due to important barriers. These might be missed with a scoping review. Although this could also simply be discussed in the limitations section.

 5) Grey literature search looks good. Do you think any additional information would be found on university/hospital websites as well?

 6) Charting, collating, and summarizing the results sections look good.

 7) The discussion section is very short. I have not reviewed a lot of study protocol manuscripts before. But it might be nice to describe what you hope to find, how the results would be helpful in more detail, and what you think the limitations would be with this approach. I would also be curious to see what you all think would be next steps afterwards. You could also state that this approach could be used as a template for other countries that might be interested in evaluating progress they have made with AMS after release of guidelines as well.

Is the study design appropriate for the research question?

Yes

Is the rationale for, and objectives of, the study clearly described?

Yes

Are sufficient details of the methods provided to allow replication by others?

Yes

Are the datasets clearly presented in a useable and accessible format?

Yes

Reviewer Expertise:

Antimicrobial stewardship, clinical research.

I confirm that I have read this submission and believe that I have an appropriate level of expertise to confirm that it is of an acceptable scientific standard, however I have significant reservations, as outlined above.

# Response

**Published:** 2024-12-12

**Authors:** Fiona Barry

**Affiliations:** School of Public Health, University College Cork, Cork, County Cork, Ireland

**Thank you for reviewing this protocol. Please see the responses to your comments below:**

**1) introduction 3rd paragraph last sentence. Reference 7 is listed at the beginning and end of the sentence.**

**Response:**

 Thank you, the first reference will be deleted

**2) This scoping review seems to focus on peer-reviewed publications to evaluate the progress of antimicrobial stewardship programs in Ireland. Is this the best way to track progress in AMS implementation? In the US, we will send out surveys to see if hospitals are meeting CDC recommendations. This may be an alternative approach to supplement published findings as well.**

**Response:**

 Thank you for your comment. We are also looking at the Grey literature in relation to identifying published evidence that actions have been taken and progress made. In addition, we plan, as a separate piece of work,  to contact hospital and community-based antimicrobial pharmacists in Ireland to assess their views on the implementation of this strategy. 

**3) I like the research objectives. It would be nice to have a table or something that outlines what the guidelines have included in 2009. I imagine you all will want to see where places are falling short of the guidelines.**

**Response:**

 Thank you, a table outlining the guidelines will be added to Stage 4.

**4) I like objective 2 as well. Some of the implementation science people will use the term facilitators and barriers to successful implementation of... You're probably looking at qualitative studies mostly here. Although I guess discussion sections of manuscripts will work well too. Again, this may also work well by being supplemented with additional survey or interview data. There might be reporting biases in publications with unsuccessful projects that may not have worked due to important barriers. These might be missed with a scoping review. Although this could also simply be discussed in the limitations section.**

**Response:**

 Thank you, this is one of the first papers in the overall research project. We will be collecting interview data in the next step. Bias will be addressed under limitations in the discussion section of the scoping review paper when published.

**5) Grey literature search looks good. Do you think any additional information would be found on university/hospital websites as well?**

**Response:**

 Thank you, in Ireland hospitals/universities do not display this information on their websites.

**6) Charting, collating, and summarizing the results sections look good.**

**Response:**

 Thank you **.**

**7) The discussion section is very short. I have not reviewed a lot of study protocol manuscripts before. But it might be nice to describe what you hope to find, how the results would be helpful in more detail, and what you think the limitations would be with this approach. I would also be curious to see what you all think would be the next steps afterwards. You could also state that this approach could be used as a template for other countries that might be interested in evaluating the progress they have made with AMS after the release of guidelines as well.**

**Response:**

 Thank you we will add:

 We will disseminate the findings through conferences, presentations, and peer-reviewed journals. These findings may inform future clinical practice and serve as a template for evaluating AMS progress at a national level, as well as for policy reform. The methodological approach may be useful in reviewing progress after the publication of AMS guidelines **.**
